# SARS-CoV-2 ORF8 accessory protein is a virulence factor

**DOI:** 10.1128/mbio.00451-23

**Published:** 2023-08-25

**Authors:** M. Bello-Perez, J. Hurtado-Tamayo, A. Z. Mykytyn, M. M. Lamers, R. Requena-Platek, D. Schipper, D. Muñoz-Santos, J. Ripoll-Gómez, A. Esteban, P. J. Sánchez-Cordón, L. Enjuanes, B. L. Haagmans, I. Sola

**Affiliations:** 1 Department of Molecular and Cell Biology, National Center for Biotechnology (CNB-CSIC), Campus Universidad Autónoma de Madrid, Madrid, Spain; 2 Viroscience Department, Erasmus Medical Center, Rotterdam, the Netherlands; 3 Veterinary Pathology Department, Animal Health Research Center (CISA), National Institute of Research, Agricultural and Food Technology, Valdeolmos, Spain; University of Hong Kong, Pokfulam, Hong Kong

**Keywords:** coronavirus, SARS-CoV-2, virulence, viral pathogenesis, accessory proteins

## Abstract

Severe acute respiratory syndrome coronavirus 2 (SARS-CoV-2) encodes six accessory proteins (3a, 6, 7a, 7b, 8, and 9b) for which limited information is available on their role in pathogenesis. We showed that the deletion of open reading frames (ORFs) 6, 7a, or 7b individually did not significantly impact viral pathogenicity in humanized K18-hACE2 transgenic mice. In contrast, the deletion of ORF8 partially attenuated SARS-CoV-2, resulting in reduced lung pathology and 40% less mortality, indicating that ORF8 is a critical determinant of SARS-CoV-2 pathogenesis. Attenuation of SARS-CoV-2-∆8 was not associated with a significant decrease in replication either in the lungs of mice or in organoid-derived human airway cells. An increase in the interferon signaling at early times post-infection (1 dpi) in the lungs of mice and a decrease in the pro-inflammatory and interferon response at late times post-infection, both in the lungs of mice (6 dpi) and in organoid-derived human airway cells [72 hours post-infection (hpi)], were observed. The early, but not prolonged, interferon response along with the lower inflammatory response could explain the partial attenuation of SARS-CoV-∆8. The presence of ORF8 in SARS-CoV-2 was associated with an increase in the number of macrophages in the lungs of mice. In addition, the supernatant of SARS-CoV-2-WT (wild-type)-infected organoid-derived cells enhanced the activation of macrophages as compared to SARS-CoV-2-∆8-infected cells. These results show that ORF8 is a virulence factor involved in inflammation that could be targeted in COVID-19 therapies.

**IMPORTANCE:**

The relevance of severe acute respiratory syndrome coronavirus 2 (SARS-CoV-2) ORF8 in the pathogenesis of COVID-19 is unclear. Virus natural isolates with deletions in ORF8 were associated with wild milder disease, suggesting that ORF8 might contribute to SARS-CoV-2 virulence. This manuscript shows that ORF8 is involved in inflammation and in the activation of macrophages in two experimental systems: humanized K18-hACE2 transgenic mice and organoid-derived human airway cells. These results identify ORF8 protein as a potential target for COVID-19 therapies.

## INTRODUCTION

Coronaviruses (CoVs) are enveloped, positive-stranded RNA viruses with a large genome (∼30 kb). To date, three highly pathogenic human CoVs have been identified, the severe acute respiratory syndrome coronavirus (SARS-CoV), Middle East respiratory syndrome coronavirus (MERS-CoV), and SARS-CoV-2, responsible for the pandemic declared in 2020 and the etiological agent of the coronavirus disease 2019 (COVID-19) (1, 2).

CoV accessory genes encode viral proteins that are not conserved among different CoV genera and are not critically essential for viral replication (3 – 5). However, they are involved in virus pathogenesis mainly by interfering with the host’s innate immune response (6, 7). Accessory proteins that behave as virulence factors are potential targets for therapeutic strategies against highly pathogenic human CoVs (8).

The number and function of accessory genes are highly variable among CoVs. MERS-CoV expresses four accessory proteins (3, 4a, 4b, and 5), while SARS-CoV-2 and SARS-CoV expresses six (3a, 6, 7a, 7b, 8, and 9b) and seven (3a, 3b, 6, 7a, 7b, 8a, and 8b), respectively (8, 9). Deletion of all accessory genes of SARS-CoV did not attenuate the virus in a human Angiotensin-Converting Enzyme 2 (hACE-2) transgenic mouse model, suggesting they might not have critical functions in virus pathogenesis (10, 5). In contrast, deletion of MERS-CoV ORF3–5 (4, 11) or ORF4b alone completely attenuated the virus in humanized human Dipeptidyl Peptidase 4 (hDPP4)-transgenic mice (12), indicating that the MERS-CoV accessory proteins are the main determinants of virus virulence.

The functions of SARS-CoV-2 accessory proteins are not fully understood. Most available studies have been performed in cancerous or transformed cell lines overexpressing individual viral proteins, outside the context of infection, thus providing results in non-physiological conditions. Among them, recent studies describe ORF6, ORF7a, ORF7b (13), ORF8 (14), and ORF9b (15) as antagonists of type I interferon (IFN-I). ORF6 was shown to inhibit STAT nuclear import and antagonize the IFN signaling pathway by its direct binding to nucleoporin Nup98 at the nuclear pore complex (16). ORF7a was described as a potent nuclear factor kappa B (NF-κB) activator of SARS-CoV-2 (17) and also an inhibitor of autophagy (18). ORF8 was shown to promote immune evasion by down-regulating the surface expression of class I major histocompatibility complex molecules (MHC-I) on several cell lines (19). Furthermore, ORF8 mimics interleukin 17A (IL-17A) and interacts with the host IL-17A receptor (IL-17RA) (20, 21), inducing a pro-inflammatory response in SARS-CoV-2-infected hamsters (22). Altogether, interactions of SARS-CoV-2 accessory proteins with mediators of the host immune response suggest a potential contribution to pathogenesis. However, to confirm their relevance in virus virulence and their impact on human disease, infections with deletion mutants of each accessory gene in physiologically relevant experimental systems, such as *in vivo* lethal animal models, or human airway organoids, are required.

In this manuscript, we engineered deletion mutants of accessory genes using our reverse genetics system for SARS-CoV-2 and evaluated their virulence *in vivo* in humanized K18-hACE2 transgenic mice. Only the deletion of ORF8 either individually (SARS-CoV-2-Δ8) or in combination with ORF6 (SARS-CoV-2-Δ[6,8]) or ORFs 6 and 7 (SARS-CoV-2-Δ[6,7,8]) partially attenuated the virus leading to 40% or 80% survival, respectively. SARS-CoV-2-Δ8 was further characterized in organoids derived from human airway epithelium. SARS-CoV-2-Δ8 attenuation was not associated with diminished replication either in the lungs of mice or in organoid-derived human airway cells. In the lungs of mice, SARS-CoV-2-∆8 induced a faster IFN response and a reduced pro-inflammatory response at late times post-infection, which could explain the attenuation. Altogether, this paper shows that SARS-CoV-2 ORF8 is a virulence factor that could be a potential target for antiviral treatment in COVID-19 patients.

## RESULTS

### Engineering deletion mutants of SARS-CoV-2 accessory genes

**Fig 1:**
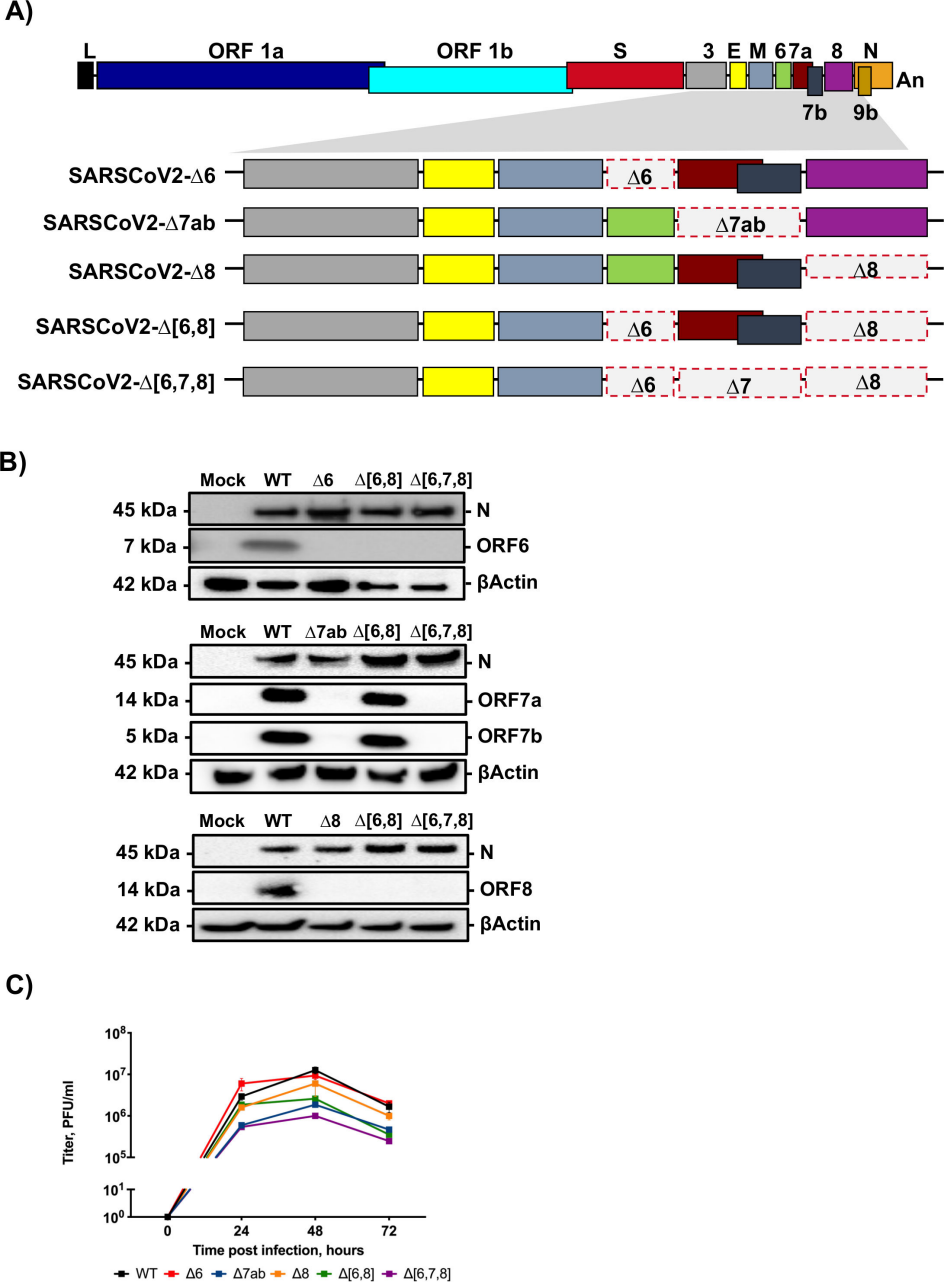
Generation and characterization of SARS-CoV-2 deletion mutants of accessory genes. (**A**) Diagram of deletions of accessory genes engineered in each mutant virus. Letters above or below boxes indicate viral genes. L, leader sequence; An, poly-A tail. Red dashed line indicates deleted regions. The shadowed area shows the 3′-end viral genes. (**B**) Immunoblot analysis of expression of accessory proteins 6, 7a, 7b, and 8 with specific antibodies in lysates from Vero E6 cells infected at an MOI 1 for 24 hours with the indicated mutants. βActin was used as a loading control. (**C**) Growth kinetics of SARS-CoV-2 deletion mutants. Vero E6/TMPRSS2 cells were infected at an MOI of 0.001, and the infections followed for 72 hours. Results are represented as a mean ± SEM.

To investigate the role of accessory genes in SARS-CoV-2 pathogenesis, recombinant viruses with individual or combined deletions of genes 6, 7ab, and 8 were engineered using a reverse genetics system previously established (23) (L. Wang, unpublished data) (Fig. 1A). All mutants were rescued and characterized in Vero E6/TMPRSS2 cells. The absence of expression of proteins encoded by the deleted genes during Vero E6/TMPRSS2 infection was confirmed by immunoblotting (Fig. 1B). The growth kinetics of mutants was evaluated at 0, 24, 48, and 72 hpi in Vero E6/TMPRSS2 cells infected at a multiplicity of infection (MOI) of 0.001 (Fig. 1C). All mutants reached titers between 10^6^ and 10^7^ PFU/mL. SARS-CoV-2-∆[6,7,8] grew to the lowest titer (10^6^ PFU/mL), suggesting that accessory genes together, although not critically essential for viral replication, were contributing to some extent to virus growth in cell cultures.

### Virulence of deletion mutants of SARS-CoV-2 accessory genes

**Fig 2:**
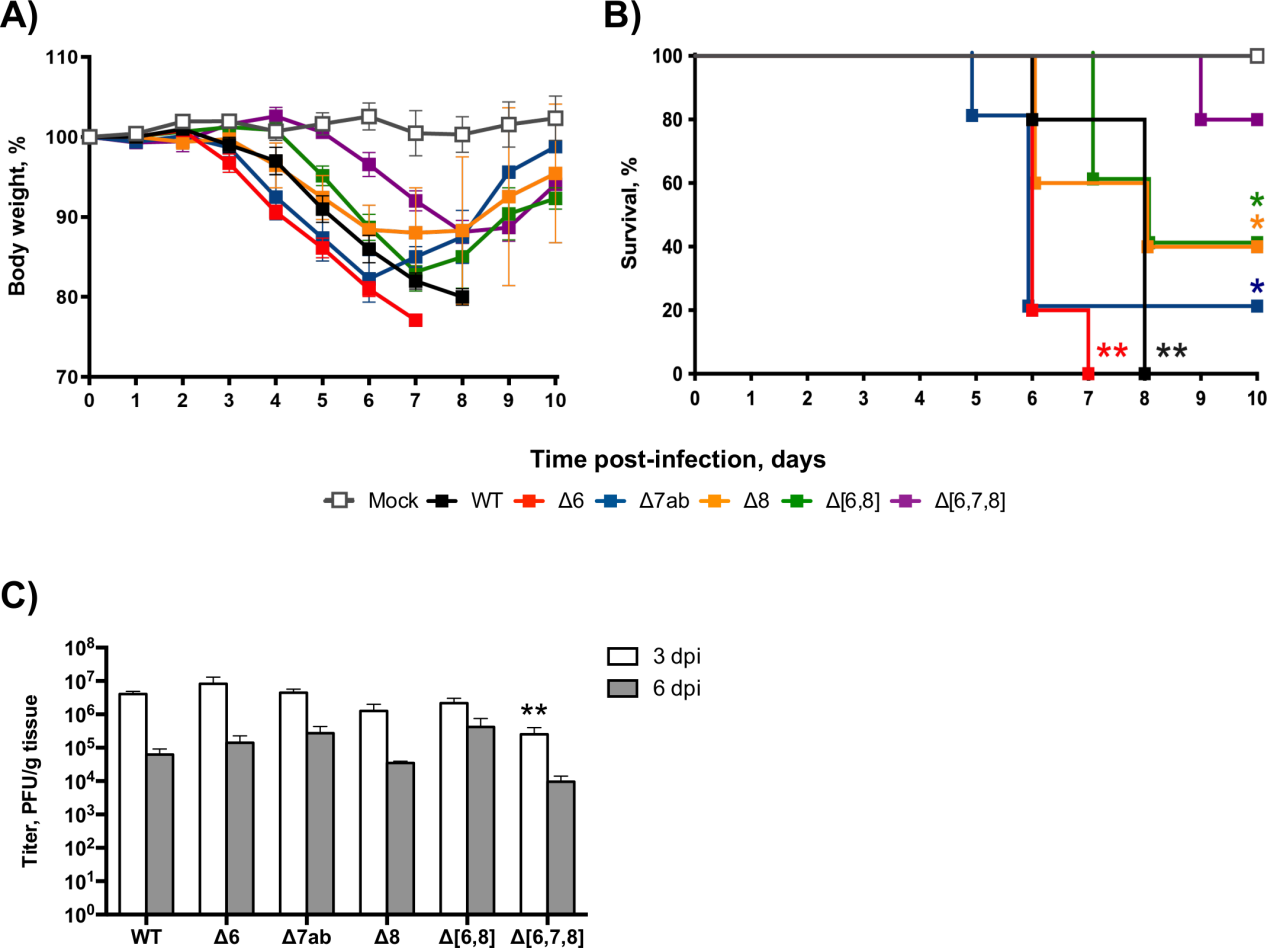
Evaluation of virulence of SARS-CoV-2 deletion mutants of accessory genes in K18-hACE2 mice. Twenty-six-week-old female K18-hACE2 mice were intranasally mock-infected or infected with 10^5^ PFU/animal of each virus. Weight loss (**A**) and survival (**B**) were monitored daily for 10 days. Weight loss is represented as the mean ± SDs of the mean (*n* = 5 mice per group). Lung samples were obtained at 3 and 6 dpi. (**C**) Viral titers were determined by plaque assays in Vero E6 cells at 4 and 6 dpi. Mean ± SDs of the mean are represented (*n* = 3). **P < 0.01; *P < 0.05.

To evaluate the relevance of each accessory protein in pathogenesis *in vivo*, 26-week-old female K18-hACE2 mice were either mock-infected, infected with SARS-CoV-2-WT (wild type), or infected with each deletion mutant (SARS-CoV-2-Δ6, SARS-CoV-2-Δ7ab, SARS-CoV-2-Δ8, SARS-CoV-2-Δ[6,8], or SARS-CoV-2-Δ[6,7,8]). Signs of clinical disease and survival were monitored daily until day 10 pi. Mock-infected mice did not lose any weight, and all of them survived. In contrast, animals infected with the deletion mutants of SARS-CoV-2 accessory genes lost weight to different extents. SARS-CoV-2-Δ6 and SARS-CoV-2-Δ7ab caused initially a severe weight loss similar to that observed in SARS-CoV-WT-infected mice, although mice infected with SARS-CoV-2-Δ7ab started recovering at 7 dpi, while all SARS-CoV-2-Δ6 died. In contrast, SARS-CoV-2-Δ8 and SARS-CoV-2-Δ[6,8] caused less severe weight losses. Initially, the weight loss caused by these viruses was tracked with that induced by SARS-CoV-2-WT, but mice recovered weight faster from 7 dpi. Infection with SARS-CoV-2-Δ[6,7,8] induced the lowest weight loss, delayed in time, starting at 6 dpi (Fig. 2A).

According to this observation, infection with SARS-CoV-2-WT, SARS-CoV-2-Δ6, or SARS-CoV-2-Δ7ab caused 80%–100% mortality, although all mice infected with SARS-CoV-2-Δ6 died 1 day before (at 7 dpi) than those infected with SARS-CoV-2-WT. In contrast, SARS-CoV-Δ8 and SARS-CoV-2-Δ[6,8] led to the death of 60% of mice (Fig. 2B). These results indicated that the deletion of ORF8, either alone or in combination with ORF6, led to a partially attenuated phenotype. The combined deletion of ORF6, ORF7ab, and ORF8 further attenuated the virus, decreasing mortality to 20%. According to log-rank test, the most virulent phenotypes (SARS-CoV-2-WT and SARS-CoV-2-∆6) show a highly significant (** *P* < 0.01) reduced survival as compared to mock-infected mice, while partially attenuated mutants (SARS-CoV-2-∆7ab, SARS-CoV-2-∆8, and SARS-CoV-2-∆[6,8]) just showed significant differences (* *P* < 0.05) in relation to mock infection. In contrast, non-significant differences in survival were observed between the highly attenuated mutant SARS-CoV-2-∆[6,7,8] and mock-infected mice. Deletion mutants replicated with titers similar to SARS-CoV-2-WT in the lungs of mice, both at 3 and 6 dpi (Fig. 2C). SARS-CoV-2-Δ[6,7,8] titers were lower than those of the WT virus at 3 dpi, although no difference was observed at 6 dpi (Fig. 2C).

**Fig 3:**
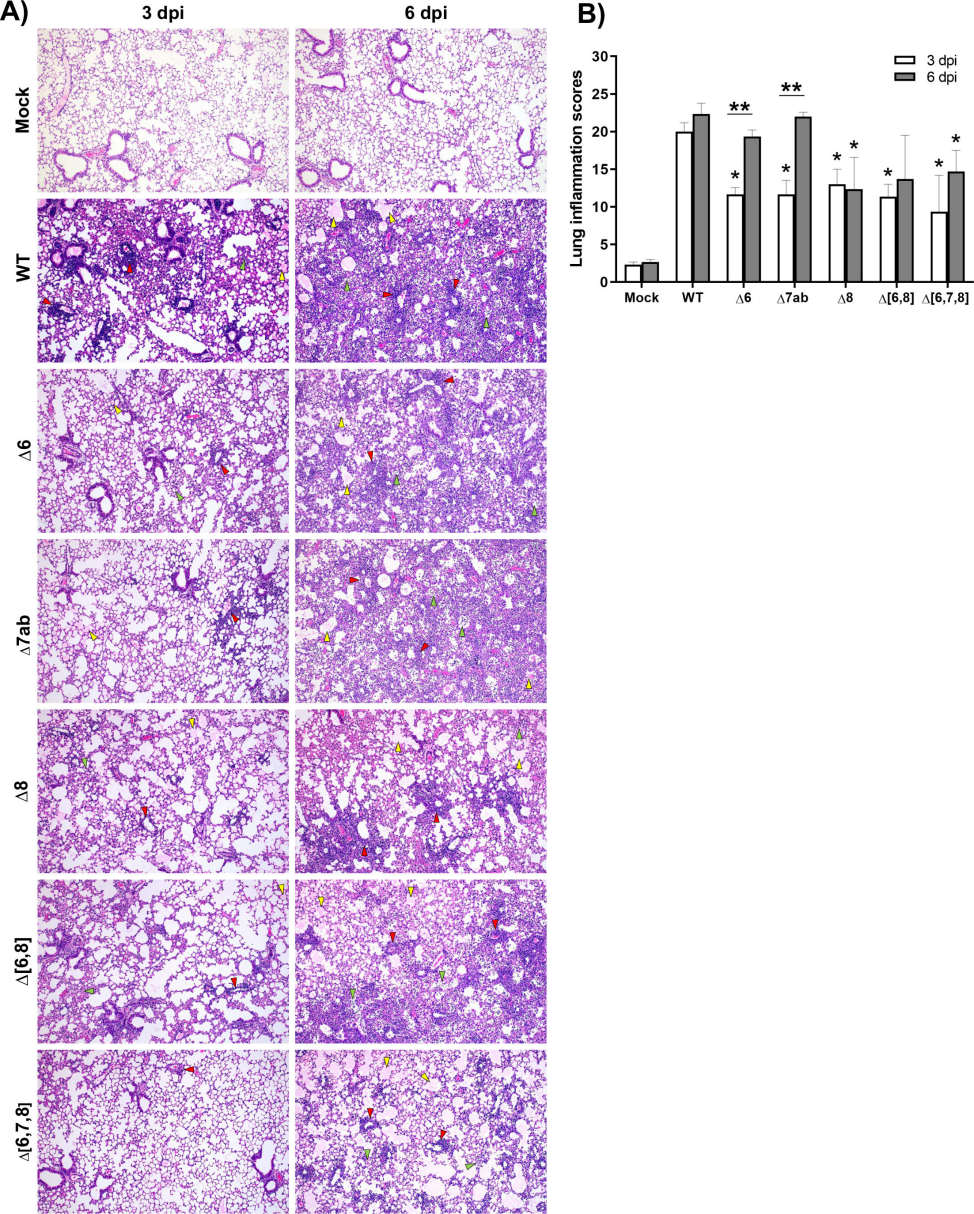
Histopathological evaluation of lungs of K18-hACE2 mice infected with SARS-CoV-2 deletion mutants of accessory genes. (**A**) Representative lung histopathological sections (H&E staining) from K18-hACE2 mice mock infected, infected with wild-type virus (WT), or infected with recombinant viruses (∆6, ∆7ab, ∆8, ∆[6,8], and ∆[6,7,8]) and euthanized at 3 and 6 dpi (magnification: 10×). Yellow arrowheads, diffuse thickening of the alveolar septae, moderate alveolar edema; green arrowheads, moderate mononuclear cell infiltrates within alveolar spaces; and red arrowheads, multifocal perivascular and peribronchiolar mononuclear infiltrates. (**B**) Lung inflammation scores examined in lung samples (left lobes) taken from K18-hACE2 mice (*n* = 3/group) intranasally mock infected, infected with wild-type virus (WT), or infected with recombinant viruses (∆6, ∆7ab, ∆8, ∆[6,8], and ∆[6,7,8]) and euthanized at 3 and 6 dpi. Mean and SEM of cumulative histopathological lesion scores. Unpaired *t*-test: *P < 0.05; **P < 0.01.

Histopathological evaluation of lungs at 3 dpi (*n* = 3/group) showed that mice infected with SARS-CoV-2-WT displayed significantly higher lung inflammation scores than mice infected with the deletion mutants (Fig. 3A and B). At 6 dpi, the lung inflammatory pathology increased up to similar levels in mice infected with the virulent viruses SARS-CoV-2-WT, SARS-CoV-2-Δ6, and SARS-CoV-2-Δ7ab. In contrast, no significant increase was observed at 6 dpi in mice infected with partially attenuated viruses SARS-CoV-Δ8, SARS-CoV-Δ(6,8), or SARS-CoV-Δ(6,7,8). Pulmonary histopathological lesions caused by the virulent viruses were severe and characterized by a diffuse thickening of the alveolar septae, massive presence of mononuclear cell infiltrates within alveolar spaces, and presence of large multifocal perivascular and peribronchiolar mononuclear infiltrates. These lesions were less severe in mice infected with partially attenuated viruses SARS-CoV-Δ8, SARS-CoV-Δ(6,8), and SARS-CoV-Δ(6,7,8) both at 3 and 6 dpi (Fig. 3A and B). As ORF8 was the only individual accessory protein with a significant contribution to SARS-CoV-2 virulence, it was selected for further analysis.

### Differences in innate immune response induced by SARS-CoV-2-WT and SARS-CoV-2-∆8

**Fig 4:**
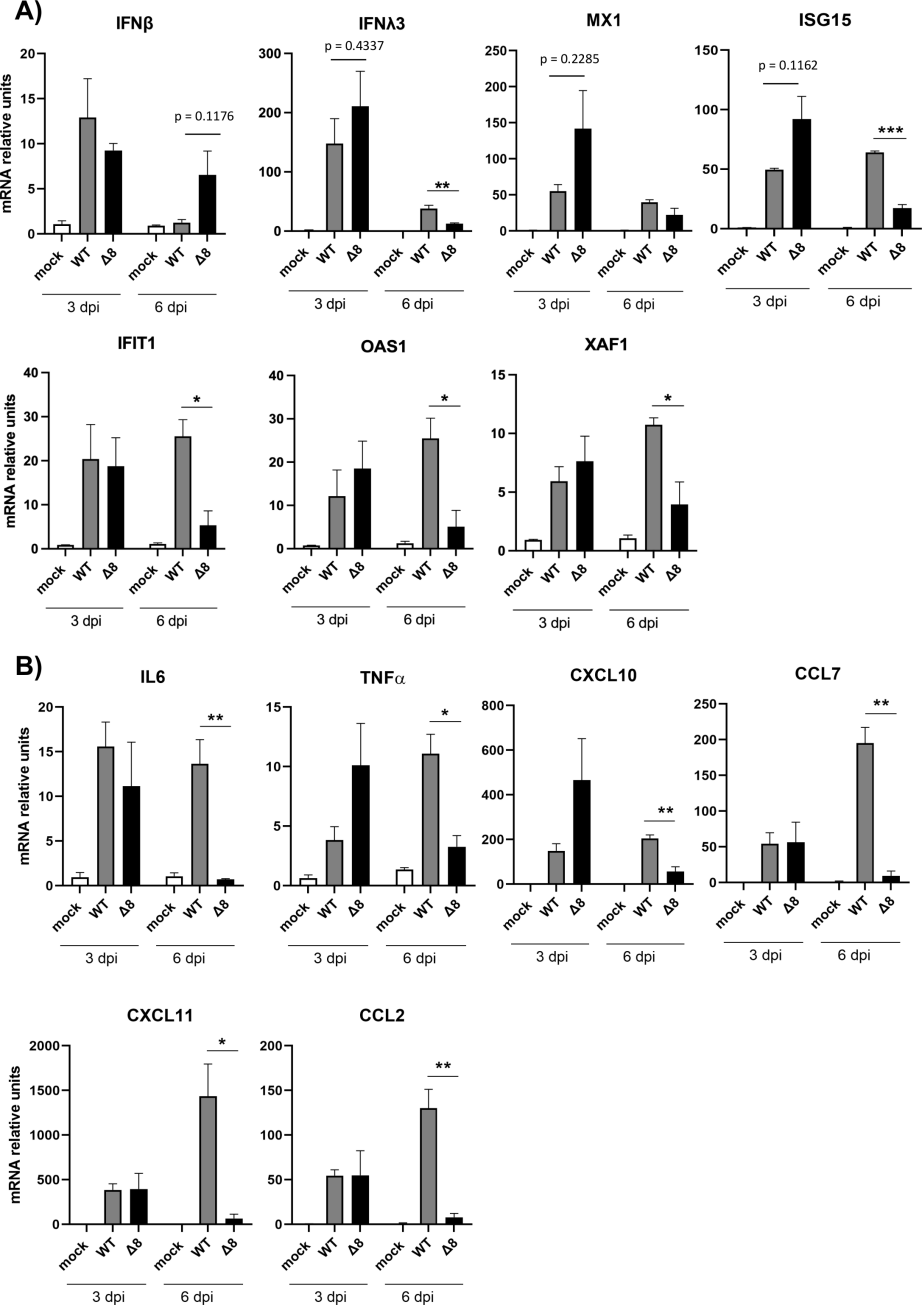
Innate immune response in the lungs of K18-hACE2 mice infected with SARS-CoV-2-WT and SARS-CoV-2-∆8. Total RNA was collected from lung samples of mice infected with WT or deletion mutant at 3 and 6 dpi. mRNAs of genes encoding interferon-related genes (IFN-β, IFN-λ3, MX1, ISG-15, IFIT1, OAS1, and XAF1) (**A**) or pro-inflammatory response genes (IL-6, TNF-α, CXCL10, CCL2, CCL7, and CXCL11) (**B**) were quantified by RT-qPCR using specific TaqMan assays. Relative mRNA levels were referred to expression in mock-infected mice. Results show means from *n* = 3 animals per group. Error bars represent SEMs. *P < 0.05; **P < 0.01; ***P < 0.001.

Early interferon responses are associated with milder symptoms in COVID-19 patients (24), while maintained elevated levels of cytokines are associated with severe COVID-19 (25). The pulmonary pathology induced by highly pathogenic human CoVs has been associated with an exacerbated pro-inflammatory response and an insufficient or delayed IFN response (3, 26 – 29). To evaluate differences in the innate immune response induced in the lungs of mice by SARS-CoV-2-WT and SARS-CoV-2-∆8 infections, a set of host mRNAs encoding interferon-related genes (IFN-β, IFNλ3, MX1, ISG15, IFIT1, OAS1, and XAF1) (Fig. 4A and B) and pro-inflammatory cytokines (IL-6, tumor necrosis factor alpha [TNF-α], chemokine [C-X-C motif] ligand [CXCL10], chemokine [C-C motif] ligand [CCL2], CCL7, and CXCL11) (Fig. 4B) were analyzed by quantitative PCR (qPCR) at 3 and 6 dpi. SARS-CoV-2-Δ8 infection showed a trend of increasing IFN signaling (IFNλ3, interferon-stimulated gene [ISG15], and MX1, *P* values 0.4337, 0.1126, and 0.2285, respectively) as compared to WT at 3 dpi, while reducing IFN-stimulated gene levels (IFNλ3, ISG15, IFIT1, OAS1, and XAF1) at 6 dpi. Similarly, the expression levels of pro-inflammatory response factors (IL-6, TNF-α, CXCL10, CCL7, CXCL11, and CCL2) were significantly decreased at 6 dpi, as compared to virulent WT infection (Fig. 4). Together, these results indicate that ORF8 contributed to promote early and time-limited IFN and pro-inflammatory responses.

**Fig 5:**
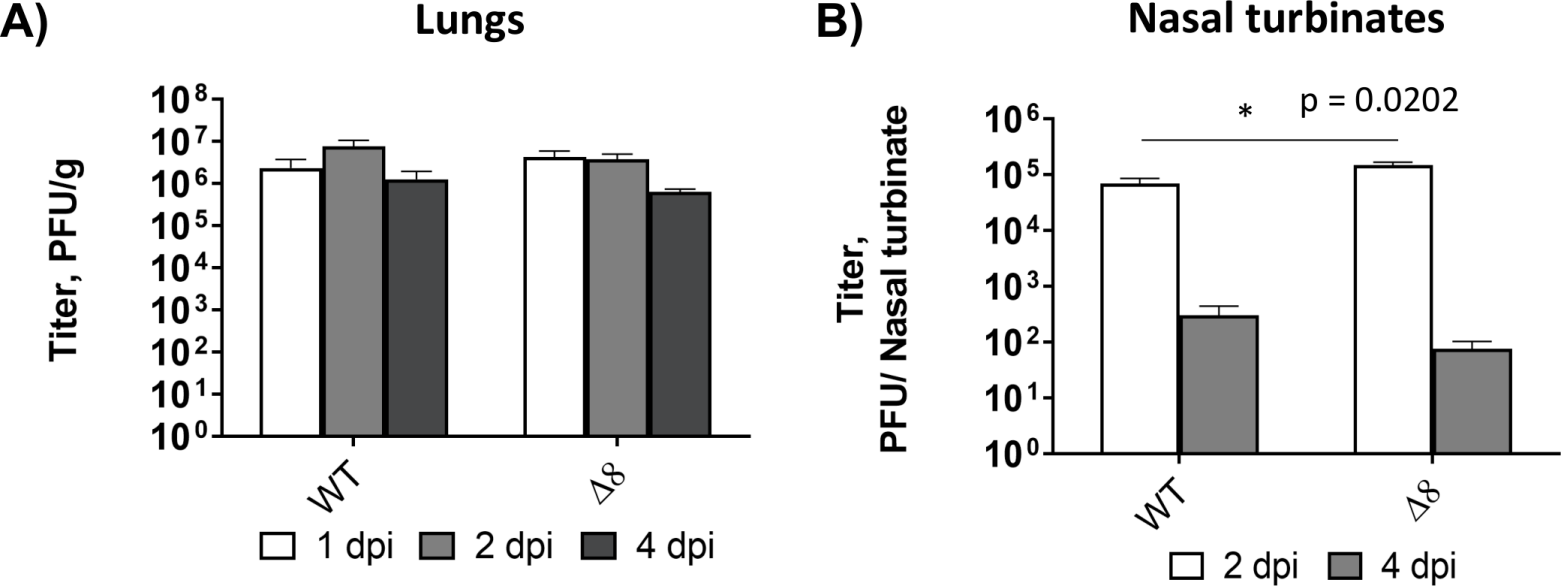
Viral titers of SARS-CoV-2-WT and SARS-CoV-2-∆8 in the lungs and nasal turbinates of infected mice. Viral titers were obtained from lungs (**A**) and nasal turbinates (**B**) samples at 1, 2, and 4 dpi. Viral titers are represented as the mean ± SEM. *P < 0.05.

**Fig 6:**
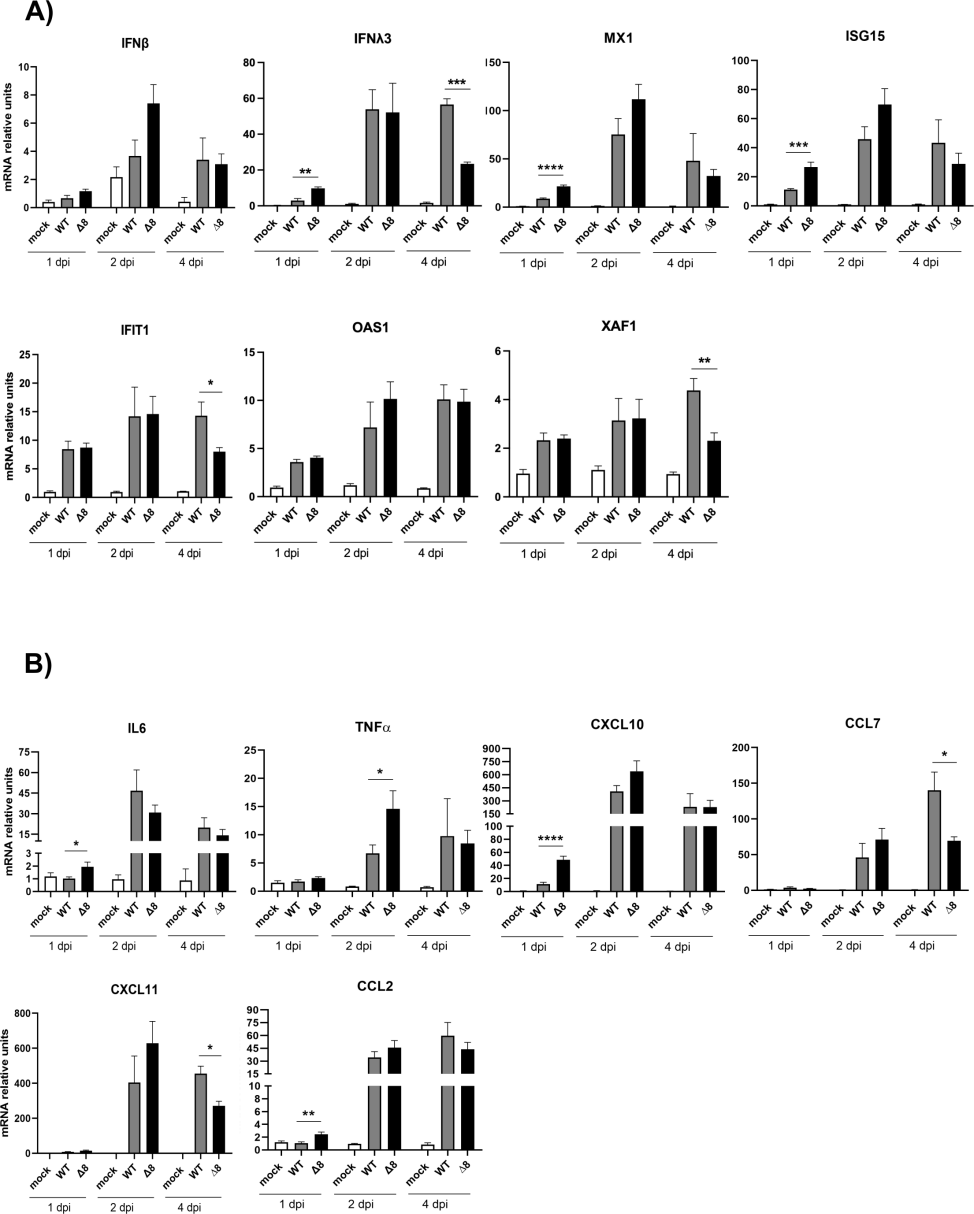
Innate immune response in the lungs of mice infected with the virulent SARS-CoV-2-WT and the attenuated mutant SARS-CoV-2-∆8 at early times post-infection. Total RNA was extracted from lung samples of mice infected at 1, 2, and 4 dpi. mRNA-encoding genes related to the interferon system (IFN-β, IFN-λ3, MX1, ISG-15, IFIT1, OAS1, and XAF1) (**A**) or the pro-inflammatory response (IL-6, TNF-α, CXCL10, CCL2, CCL7, and CXCL11) (**B**) were quantified by RT-qPCR using specific TaqMan assays. Relative mRNA levels were based on comparison with mock-infected mice. Results show means from *n* = 3 animals per group. Error bars represent SEMs. *P < 0.05; **P < 0.01; ***P < 0.001; ****P < 0.001.

To confirm whether the temporal regulation of interferon and pro-inflammatory responses was associated with SARS-CoV-2-∆8 attenuation, a second experimental infection of mice was performed at earlier time points. Similar levels of infectious SARS-CoV-2-WT and SARS-CoV-2-∆8 were detected in the lungs of mice at 1, 2, and 4 dpi (Fig. 5A). Minimal differences in viral growth were also observed in the nasal turbinates of mice (Fig. 5B). mRNA expression levels of IFN-related genes (Fig. 6A) and pro-inflammatory cytokines (Fig. 6B) were measured in the lungs of mice at 1, 2, and 4 dpi. As compared to SARS-CoV-2-WT, SARS-CoV-2-∆8 induced higher expression levels at 1 or 2 dpi, which decreased at 4 dpi to become lower than those of the SARS-CoV-2-WT (Fig. 6). These results suggested that the combination of early IFN responses and the reduction of an inflammatory state at late times post-infection contributed to the partial attenuation of SARS-CoV-2-∆8.

**Fig 7:**
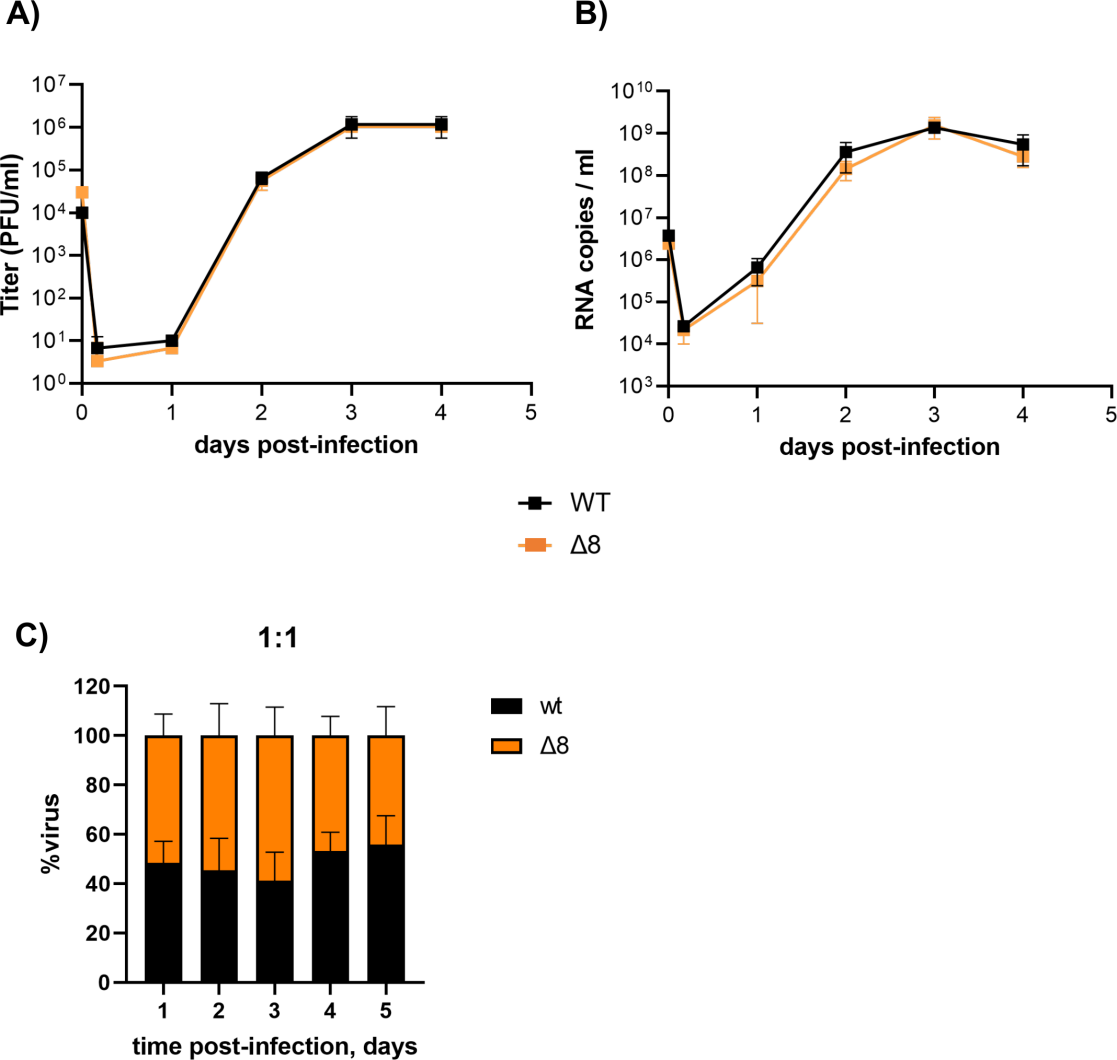
Characterization of SARS-CoV-2-WT and SARS-CoV-2-∆8 in two-dimensional (2D) human airway organoids. Representative data of replication kinetics in terms of viral titers (**A**) and RNA (**B**) studied in organoids from three different donors. (**C**) Proportion of replication of each virus in 2D airway organoids infected with a 1:1 ratio.

To confirm that ORF8 was also a virulence factor in human cells, SARS-CoV-2-∆8 mutant was characterized in organoid-derived human airway cultures, previously used to study respiratory virus infections, including SARS-CoV-2 (30, 31). In these organoids, basal cells can be differentiated at air–liquid interface (ALI) into a reconstituted airway culture containing basal, goblet, club, and ciliated cells. In this model, ciliated cells are susceptible to SARS-CoV-2 infection, just as observed in humans (30, 31). Both SARS-CoV-2-WT and SARS-CoV-2-∆8 grew to similar, relatively high titers on these cells (~10^6^ PFU/mL), without significant differences in the kinetics, as shown by plaque-assay titration in VeroE6 cells (Fig. 7A) and viral RNA quantification (Fig. 7B). In addition, competition assays by co-infecting organoids with similar proportions (1:1) showed that both viruses had similar replication fitness (Fig. 7C).

**Fig 8:**
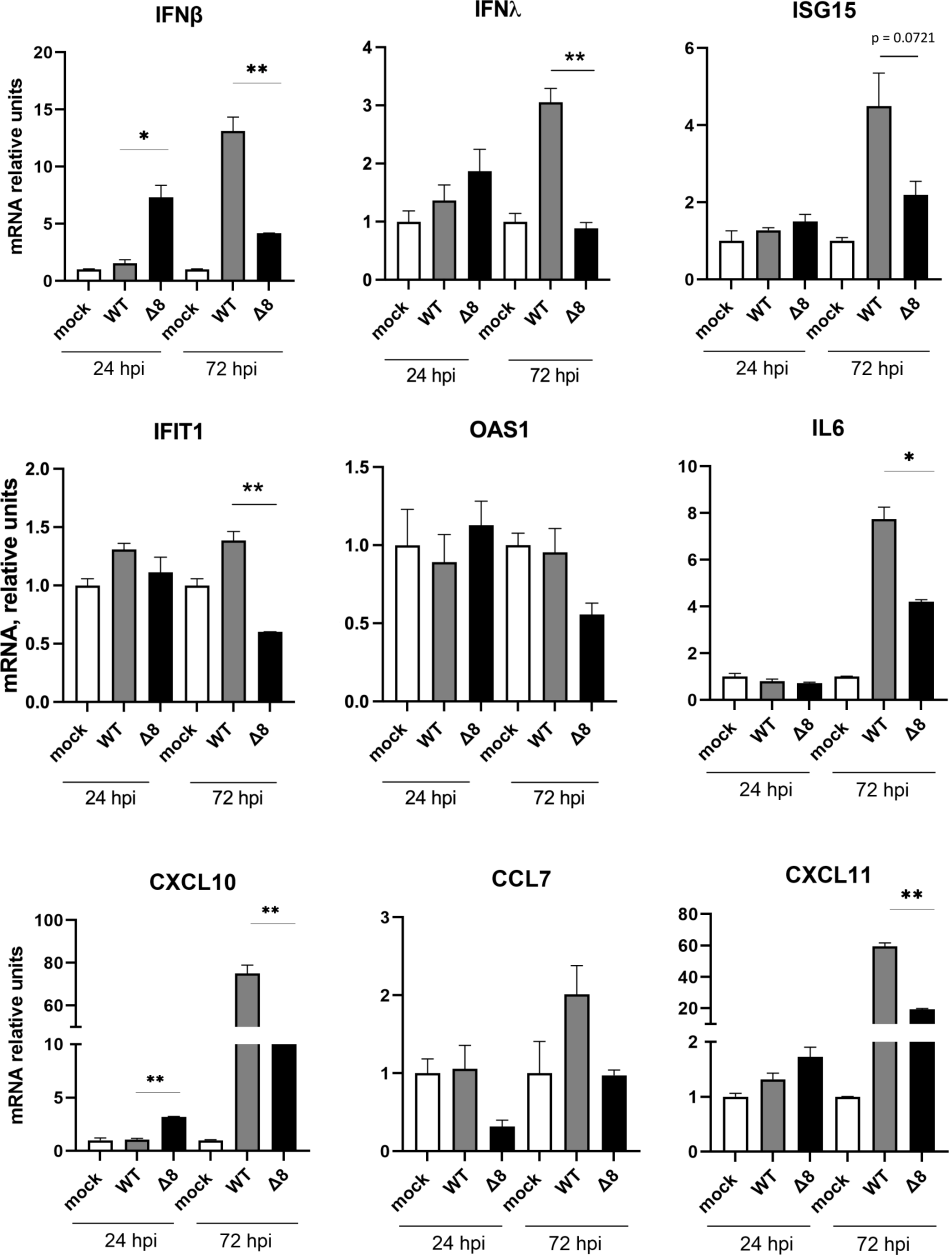
Innate immune response in organoid-derived human airway cells infected with SARS-CoV-2-∆8 and SARS-CoV-2. Total RNA was extracted from cells infected with WT or deletion mutant at 24 and 72 hpi. mRNA-encoding genes related to the interferon system (IFN-β, IFN-λ, ISG-15, IFIT1, and OAS1) (**A**) or the pro-inflammatory response (IL-6, CXCL10, CXCL11, and CCL7) (**B**) were quantified by RT-qPCR using specific TaqMan assays. Relative mRNA levels were based on comparison with mock-infected mice. Results show means from *n* = 2 donors. Error bars represent SEMs. *P < 0.05; **P < 0.01; ***P < 0.001.

To confirm the effect of ORF8 on the innate immune response in a human cell model, organoid-derived human airway cells were infected with SARS-CoV-2-WT or SARS-CoV-2-∆8. Infectious viruses and intracellular RNA were collected at 24 and 72 hpi. In accordance with the *in vivo* results, SARS-CoV-2-∆8 infection induced higher expression levels of IFN-β and pro-inflammatory cytokines than SARS-CoV-2-WT at 24 hpi and lower levels of IFN-β, IFN-λ, and IFIT1 at 72 hpi (Fig. 8). These results support a potential mechanism of attenuation of SARS-CoV-2-∆8 by inducing early and time-limited innate immune responses, in contrast to more delayed and sustained responses induced by the virulent SARS-CoV-2-WT.

### Differences in the cellular immune response induced by SARS-CoV-2-WT and SARS-CoV-2-∆8

**Fig 9:**
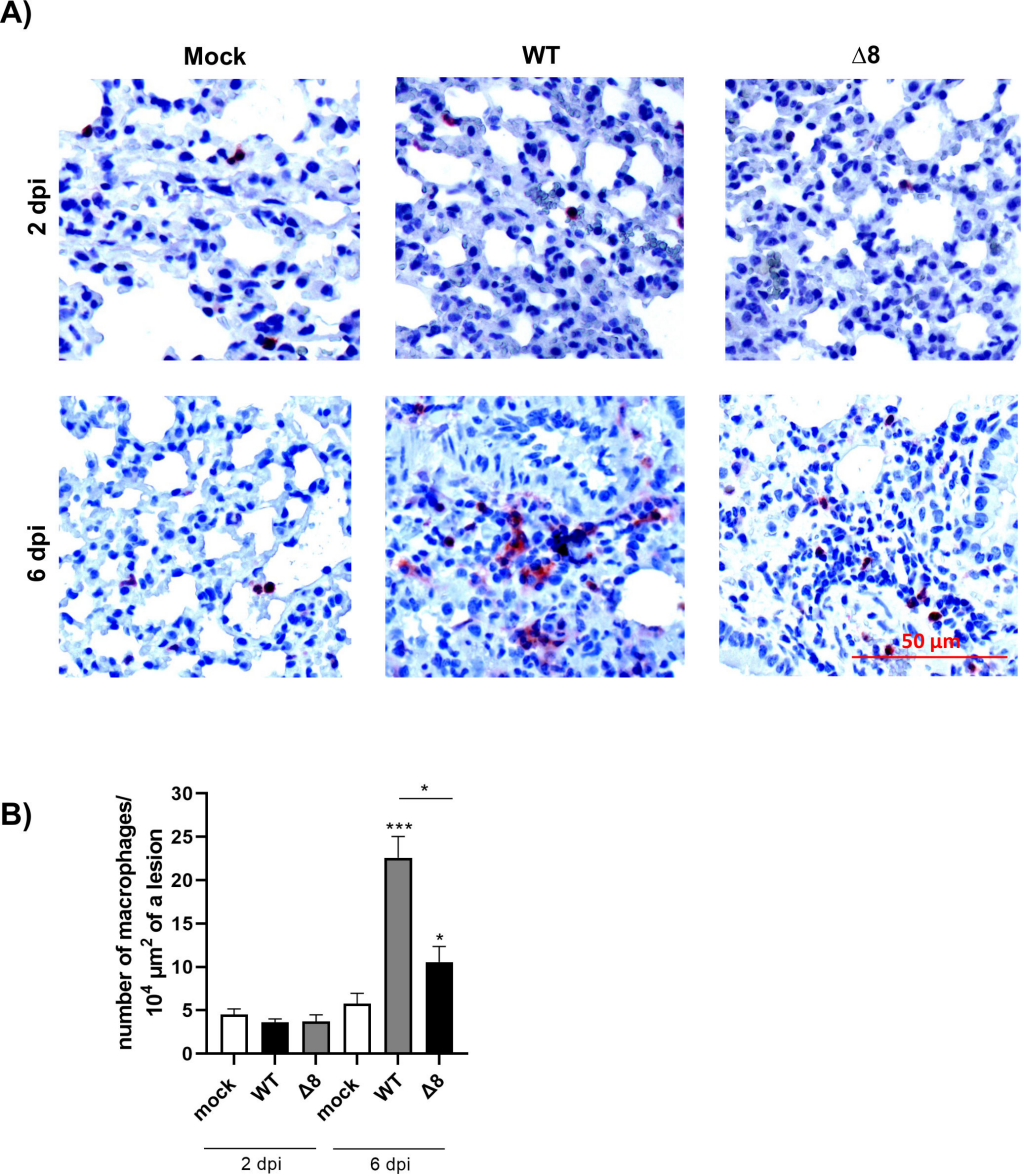
Immunohistochemical stainings for macrophages in mice lungs. (**A**) Representative images of pulmonary macrophages observed in mice euthanized at 2 and 6 dpi. (**B**). Quantification of macrophages. Bars represent means of three mice. Error bars represent SEMs. *P < 0.05; **P< 0.01; ***P < 0.001.

Macrophages are immune cells with relevant functions in host defense. Since their dysregulated hyperinflammation has also been proposed to contribute to SARS-CoV-2 pathogenesis (32), we studied macrophage responses in the lungs of K18-hACE2 mice and in the organoid model after SARS-CoV-2-WT and SARS-CoV-2-∆8 infection. Notably, the number of macrophages was significantly increased in infected mice at 6 dpi. However, this increase was higher in mice infected with SARS-CoV-2-WT than in those infected with SARS-CoV-∆8 at 6 dpi (Fig. 9A and B). Moreover, in the lesions of SARS-CoV-2-WT-infected mice, macrophages aggregated into small clusters at 6 dpi. In contrast, a more diffuse distribution of macrophages was observed in SARS-CoV-2-∆8-infected mice (Fig. 9A).

**Fig 10:**
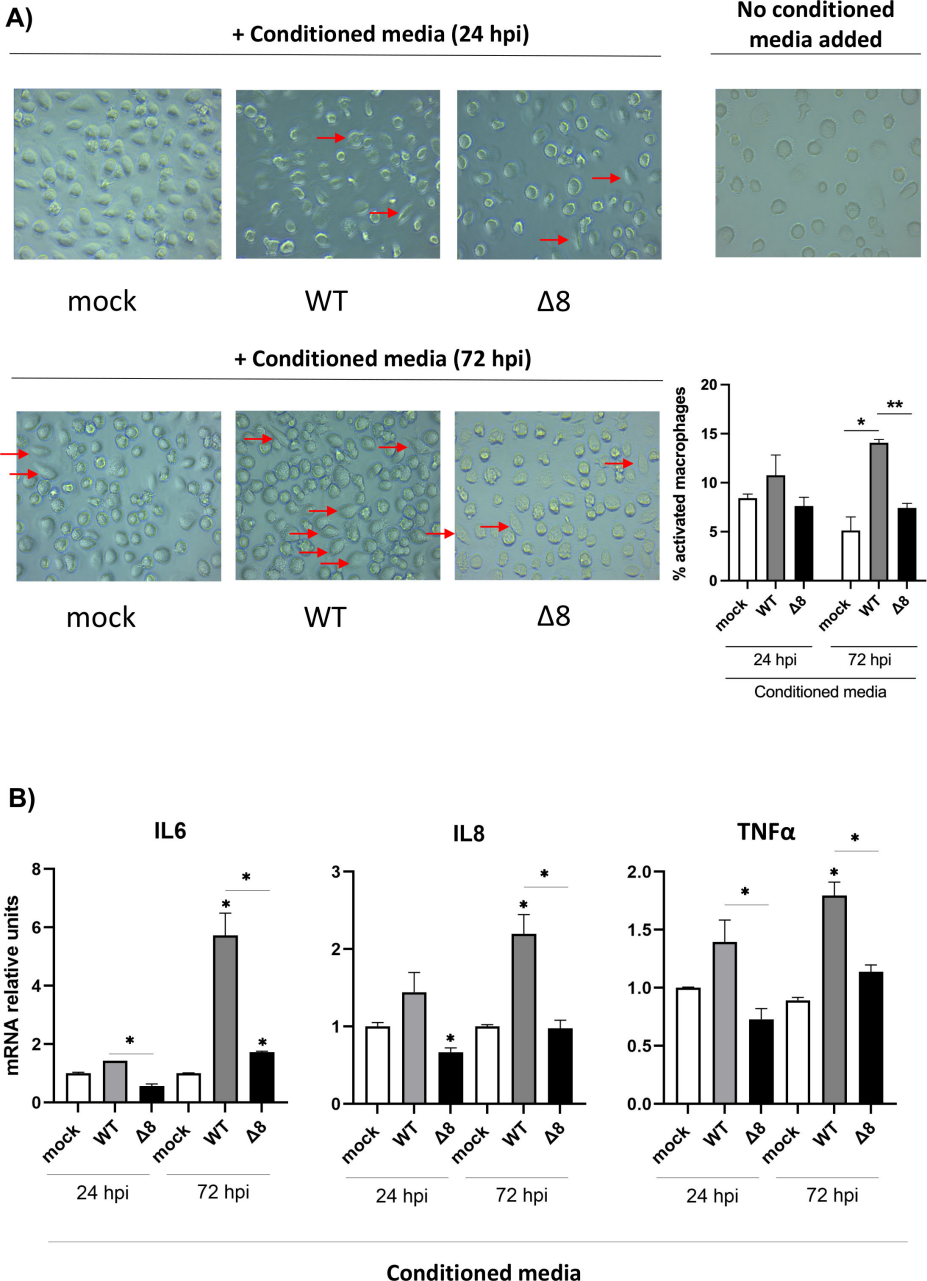
Evaluation of human macrophages’ activation. (**A**) Human macrophages were treated with conditioned media from mock-infected, infected with SARS-CoV-2-WT or SARS-CoV-2-∆8 organoids for 24 hours, and the cellular morphology was analyzed with a microscope. Activated macrophages were marked with arrows. (**B**) Quantitative reverse transcription PCR (RT-PCR) analysis of the relative expression of IL-6, IL-8, and TNF-α mRNA. Error bars represent SEMs. *P < 0.05; **P < 0.01; ***P < 0.001.

The activation of human macrophages was evaluated using the organoid-derived human airway cells. Human macrophages were incubated with conditioned media collected from the basal compartment of infected organoids at 72 hpi, which contains activating cytokines. Macrophage activation was observed as a morphological transformation from round-shaped cells to flattened spreading cells with pseudopodium-like protrusions (Fig. 10A). A higher number of activated cells was observed when macrophages were incubated with conditioned media obtained after 72 hpi from organoids infected with SARS-CoV-2-WT, as compared to the media from SARS-CoV-2-∆8-infected organoids. Accordingly, mRNA levels of IL-6, IL-8, and TNF-α were significantly higher when macrophages were activated by the conditioned media from organoids infected with SARS-CoV-2-WT (Fig. 10B). These results suggest that macrophages could be crucial to promote the hyperinflammation observed in patients with critical disease manifestations since over-activation of these cells could result in a cytokine storm.

## DISCUSSION

CoV accessory genes play important roles in pathogenesis (4, 33, 34). However, the relevance of SARS-CoV-2 accessory genes in virulence still remains unclear, since limited information is available from studies performed in the context of SARS-CoV-2 infection in animal models (9). Using a SARS-CoV-2 reverse-genetics system to engineer infectious cDNAs with deletions of genes, we studied the relevance of accessory genes to the virus pathogenesis in the K18-hACE2 mouse model. The individual deletion of ORF6 and ORF7ab did not significantly affect SARS-CoV-2 virulence, suggesting a minor contribution to the pathogenesis of these genes separately. However, the deletion of ORF8, either alone or in combination with ORF6, partially attenuated SARS-CoV-2 by reducing the weight loss, lethality, and lung inflammatory pathology of infected mice, indicating that ORF8 is a virulence factor. The highest attenuation was observed by the combined deletion of the four accessory ORFs 6, 7a, 7b, and 8, suggesting that the deletion of ORF7ab in the absence of ORFs 6 and 8 further attenuates SARS-CoV-2.

The most characteristic pulmonary histopathological lesions induced by WT and virulent mutants (SARS-CoV-2-Δ6 and SARS-CoV-2-Δ7ab) included severe diffuse thickening of the alveolar septae, severe diffuse mononuclear cell infiltrates within alveolar spaces as well as the presence of large multifocal perivascular and peribronchiolar mononuclear infiltrates (35, 36). In contrast, the lungs infected with partially attenuated SARS-CoV-2-Δ8, SARS-CoV-2-Δ[6,8], and SARS-CoV-Δ[6,7,8] showed only mild to moderate lung damage. In addition, a reduction in the number of macrophages was observed in lung lesions in mice infected with SARS-CoV-2-Δ8 compared to lungs infected with SARS-CoV-2-WT at late times post-infection, previously associated with mild SARS disease in BALB/c mice (37). An excessive macrophage response could induce abnormal secretion of pro-inflammatory factors causing apoptosis of endothelial and epithelial cells of the lungs, resulting in hypoxia, alveolar edema, and vascular leakage (38).

Natural variants with mutations in ORF6 (39, 40), ORF7a (41, 42), ORF7b (43), or ORF8 (43 – 45) have been described. ORF6 variants showed no differences in clinical presentations as compared to hospitalized patients infected with SARS-CoV-2-WT (40). However, variants with mutations in ORF8, either a large 382-nt deletion comprising part of 7b and ORF8 transcription-regulating and coding sequences (45) or the alpha variant (B1.1.7), with a stop mutation at Q27 (46), were associated with a milder COVID-19 disease, suggesting that ORF8 might contribute to SARS-CoV-2 virulence. Since these natural variants include a variety of mutations in addition to those in ORF8, it is not possible to establish a causal relationship between the expression of ORF8 and the severity of the disease. However, these findings support a potential contribution of ORF8 to the virulence of SARS-CoV-2, as proposed by one study showing that ORF8 mimics the host IL-17 cytokine and contributes to COVID-19 severe inflammation (21) and the observation that SARS-CoV-2-∆8 induces less inflammation in the lungs of hamsters (22). In addition, a histone mimic motif was described in ORF8 (47). Chromatin remodeling induced by viral infections can disrupt epigenetic regulation of gene expression, including the immune response, which might contribute to pathogenicity. However, the implications of this host response in acute infection or post-acute sequelae of SARS-CoV-2 are not well understood yet.

In contrast, another study postulated that ORF8 plays a minor role in the disease outcome since the deletion of ORF8 from the SARS-CoV-2 infectious cDNA induced 100% of mortality in K18-hACE2 mice (9). Experimental differences in the age of mice between that study (4–8 weeks old) (48) and our work (26 weeks old) might explain the differences observed, as it is well known that SARS-CoV-2 virulence is age dependent. Moreover, using the hamster sublethal model, one study described that infection with SARS-CoV-2 deficient in ORF6 showed significantly reduced weight loss as compared to WT-infected animals. However, both viruses caused in the lungs similar histopathological lesions, including necrosuppurative bronchiolitis at 2 dpi that progressed to bronchointerstitial pneumonia with edema and hemorrhage at 4 dpi, suggesting that ORF6 was not a major determinant of virulence (49).

The individual deletion of SARS-CoV-2 accessory genes did not affect viral titers in cell cultures, in the organoid model, or in susceptible mice, supporting the idea that they are not essential for replication, as demonstrated for other coronaviruses, SARS-CoV and MERS-CoV (4, 5, 9). The combined deletion of the four accessory genes (6, 7a, 7b, and 8) of SARS-CoV-2 led to a slight reduction in viral titers both in VeroE6/TMPRSS2 cells and *in vivo*. Similarly, the attenuated deletion mutant MERS-CoV-Δ[3,4a,4b,5] showed a reduction in viral replication (4, 50). Conversely, attenuation of MERS-CoV by the deletion of single accessory genes is mainly caused by the induction of less inflammation and not by lower viral titers in the lungs (4, 12).

It is well-documented that CoVs encode multiple interferon antagonists to evade the host innate immune response (51 – 53). Accessory proteins are not conserved among CoVs and have different functions, mostly related to the interference with the innate immune response. Highly pathogenic human CoVs induce a modest IFN response (26), as confirmed in this study (Fig. 3A and Fig. 4A), which is associated with disease severity and depends on the expression of viral genes that interfere with the host antiviral response. Deletion of ORF8 increased the early expression of MX1 and ISG15 at 1 dpi in the lungs of mice and IFNβ in human organoids at 24 hpi, suggesting that ORF8 is an IFN antagonist at early times post-infection. Overexpression of SARS-CoV-2 ORF8 in HEK-293 T cells led to controversial results. Studies described that ORF8 downregulated the expression of IFN-β and ISGs induced by Sendai virus infection (14) or poly I:C (54) by decreasing nuclear import of IRF3, while in another study ORF8 did not suppress IFN production or signaling (55). These different results might be explained by the overexpression of a single protein at non-physiological levels in the absence of other viral proteins. Moreover, preferably all the results obtained relating one protein with one viral function should be validated in physiologically relevant infection models, such as human organoids, as this work reported. In fact, the organoid model used here is useful to study the virulence of SARS-CoV-2 as it recapitulates the lung inflammatory disease observed in transgenic mice and humans (56, 57).

The early increase in the production of IFN and the down-regulation of pro-inflammatory cytokines at late times post-infection with SARS-CoV-2-Δ8 led to a partial attenuation (Fig. 2, 3, 4 and 6), suggesting a protective effect, as previously described for the early administration of IFN in SARS-CoV- (37) and MERS-CoV- (58) infected mice. The increase of IFN at late times post-infection has been associated with excessive recruitment of monocytes and with a more severe infection in mice in the case of SARS-CoV (37). In the same line, COVID-19 patients with milder symptoms induced higher levels of interferon early in infection and lower levels of pro-inflammatory cytokines than severe patients (56, 57). In contrast, prolonged IFN responses resulted in a delayed adaptive immune response and continued upregulation of inflammatory chemokines (59). In accordance with this observation, the SARS-CoV-2-Δ382 isolate lacking ORF8 expression induced higher levels of IFN, but lower levels of pro-inflammatory responses, resulting in a milder infection (45).

Together, these results support that ORF8 promotes an innate immune response to a viral infection that contributes to immune-mediated pathology. A slight increase in IFN at early times post-infection, not prolonged in time, and a decrease in the pro-inflammatory responses during infection likely protected 40% of mice from death and reduced macrophage recruitment and activation. These results provide evidence for the relevance of innate immune timing in inflammatory pathology and disease outcome. Moreover, since ORF8 is a virulence factor during SARS-CoV-2 infection, therapies targeting ORF8 might contribute to limit immunopathology and improve the disease outcome.

## MATERIALS AND METHODS

### Cells

African green monkey kidney-derived Vero E6 cells were kindly provided by Dr. Eric Snijder (Leiden University Medical Center, the Netherlands). Vero E6 cells stably expressing TMPRSS2 (Vero E6/TMPRSS2 cells) were obtained from the American Type Culture Collection (ATCC). Cells were grown in Dulbecco-modified Eagle’s medium (DMEM; Gibco, United Kingdom) supplemented with 25 mM HEPES (Gibco), 50 µg/mL gentamicin (Sigma-Aldrich, USA), 2 mM l-glutamine (Sigma-Aldrich), 1% (vol/vol) non-essential amino acids (Sigma-Aldrich), and 10% (vol/vol) HyClone fetal bovine serum (FBS; Gibco). Vero E6/TMPRSS2 was supplemented with 1 mg/mL G418 (Sigma-Aldrich). Infected cells were maintained in DMEM supplemented with 2% FBS. Vero E6/TMPRSS2 cells were used for the rescue of infectious viruses from cDNA and to characterize virus growth, while Vero E6 cells were used for viral titration.

### Organoid culture and differentiation

Human adult airway stem cells were isolated from lung parenchyma (*n* = 2) and grown as described previously (30). Adult human lung tissue was obtained from non-tumor lung tissue from patients undergoing lung resection. The Medical Ethical Committee of the Erasmus MC granted permission for this study (METC 2012-512). Stem cells were isolated from adult lung tissue, maintained, and passaged in Basement Membrane Extract (BME) in the airway organoid medium described previously (30). To obtain cultures for infection experiments, stem cells were dissociated into single cells and seeded on Transwell membranes. Cells were differentiated at ALI in Pneumacult-ALI medium (StemCell Technologies) for at least 4 weeks before performing infection experiments, and the medium was replaced every 5 days.

### Plasmids and bacterial strains

Bacterial artificial chromosome (BAC) pBeloBAC1 was used to assemble recombinant SARS-CoV-2 infectious cDNA clones. BAC plasmids were purified using a large-construct kit (Qiagen), following the manufacturer’s specifications. *Escherichia coli* DH10B (Invitrogen, Thermo Fisher Scientific) cells were transformed by electroporation using a MicroPulser unit (Bio-Rad) according to the manufacturer’s instructions.

Methylase-negative *dam^-^/dcm^-^ E. coli* bacteria (New England Biolabs, USA) were used to amplify several plasmids [pSL-F6, pUC57-F∆7ab, and pUC57-∆(6,7,8)] to yield DNA susceptible to cleavage by methylation-sensitive PpuMI.

### Construction of plasmids including the cDNA of SARS-CoV-2 deletion mutants

pBAC-SARS-CoV-2-FL plasmid, including the full-length cDNA of SARS-CoV-2 (GenBank accession no. MN908947.3), was used as the basis to engineer mutants with individual or combined deletions of accessory genes: SARS-CoV-2-∆6, SARS-CoV-2-∆7ab, SARS-CoV-∆8, SARS-CoV-2-∆[6,8], and SARS-CoV-2-∆[6,7,8].

Deletions of ORF6, ORF7ab, ORF8, and ORFs (6,7,8) were carried out using intermediate plasmids, including chemically synthesized cDNA fragments of the SARS-CoV-2 genome with the deletions of interest, generated by GenScript (Thermo Fisher Scientific). pUC57-FΔ6-contained fragment ∆6 (F∆6), which included the SARS-CoV-2 sequence comprised between nt 27,009 and 27,612 with a 166-nt deletion corresponding to ORF6 (nt 27,202–27,367). pUC57-FΔ7ab comprised nt 27,009–28,609 of the SARS-CoV-2 with a 372-nt deletion (nt 27,388–27,759) corresponding to ORF7ab. pUC57-FΔ8 contained SARS-CoV-2 nt 27,613–28,609 with a 353-nt deletion corresponding to ORF8 (nt 27,888–28,240). pUC57-F∆(6,7,8) contained SARS-CoV-2 nt 27,009–28,609 with a 1,038-nt deletion corresponding to ORFs(6,7,8) (nt 27,202–28,240).

Viral sequences from pUC57-FΔ6, pUC57-FΔ7ab, pUC57-FΔ8, and pUC57-FΔ(6,7,8) plasmids flanked by specific restriction sites were subcloned into the intermediate plasmid pSL-F6, which contained the 3′-end sequence of the SARS-CoV-2 genome (nt 25,314–28,609). pUC57-F∆6 and pUC57-F∆eight were digested with BmgBI and PpuMI restriction enzymes, while pUC57-F∆7ab and pUC57-∆(6,7,8) were digested with PpuMI and AvrI. The resulting fragments were subcloned into the same sites of pSL-F6. Then, the AvrII-ApaLI fragments with the deletion of accessory genes were transferred from pSL-∆6, -∆7ab, -∆8, and -∆(6,7,8) plasmids to pBAC-F6, a pBeloBAC1 plasmid containing 3′-end sequence of SARS-CoV-2 from nt 25,314. The resulting plasmids pBAC-F6-∆6, pBAC-F6-∆7ab, pBAC-F6-∆8, and pBAC-F6-∆[6,7,8] were digested with BamHI and RsrII, and the fragments carrying the deletion were inserted into the pBAC-SARS-CoV-2-FL digested with the same enzymes.

To delete the ORF6 and ORF8 in combination, pUC57-F∆8 was digested with BmgBI and AvrII, and the insert, with the ORF8 deletion, was cloned into the same site of pSL-F6-∆6. The resulting plasmid pSL-F6-∆(6,8) was digested with AvrII and ApaLI, and the insert was subcloned into the intermediate plasmid pBAC-F6. Finally, the plasmid pBAC-F6-∆(6,8) was digested with BamHI and RsrII, and the viral sequence with ORF6 and ORF8 deleted was cloned into pBAC-SARS-CoV-2-FL.

### Transfection and recovery of infectious SARS-CoV-2 deletion mutants from cDNA clones

To recover recombinant infectious viruses, Vero E6/TMPRSS2 cells grown in 12.5-cm^2^ flasks at 95% confluence were transfected with 6 µg of each infectious cDNA clone using Lipofectamine 2000 (Invitrogen, Thermo Fisher Scientific), according to manufacturer’s specifications. After 72 hours of incubation at 37°C, supernatants were harvested, passaged once on fresh Vero E6/TMPRSS2 cells, and then virus titers were determined by plaque assay in Vero E6 cells, as described below. Viral stocks for *in vivo* and *in vitro* evaluation were produced by harvesting supernatants from passage 1. Supernatants were stored at −80°C. The complete genomic sequence of the recombinant viruses was verified by sequencing cDNA fragments synthesized by RT-PCR.

### Viral titration

Virus titers were determined by plaque assays on Vero E6 cells grown to 100% confluence, using plates placed in sealed plastic bags. Briefly, after 1 hour of absorption with serial dilutions of viruses at 37°C, the medium was removed and cells were overlaid with either DMEM supplemented with 4 mM glutamine, 1% vol/vol non-essential amino acids, 2% vol/vol FBS, 0.16 mg/mL of DEAE-Dextran and 1% low-melting agarose or Opti-MEM I (1×) + GlutaMAX (Gibco), and 1.2% Avicel (FMC biopolymers). After 48–96 hours, cells were fixed with 10% formaldehyde and either stained with 0.1% (wt/vol) crystal violet in 20% methanol or blocked in 3% bovine serum albumin (BSA; Sigma-Aldrich) followed by primary antibody staining (rabbit anti-nucleocapsid; Sino Biological) in 0.6% BSA for an hour and secondary antibody staining (goat anti-rabbit Alexa Fluor 488; Invitrogen) in 0.6% BSA for an hour. Plates stained by immunofluorescence were scanned on the Amersham Typhoon Biomolecular Imager (GE Healthcare) and analyzed using ImageQuant TL 8.2 software (GE Healthcare). Titers were determined by multiplying the number of PFUs by the dilution factor and referred to a volume of 1 mL (PFU/mL), grams of lung tissue (PFU/g), or the whole nasal turbinate (PFU/nasal turbinate).

### Growth kinetics

Confluent monolayers of Vero E6/TMPRSS2 cells grown in 24-well plates were infected with SARS-CoV-2 deletion mutants at an MOI of 0.001 PFU/cell. Cell supernatants were collected at 0, 24, 48, and 72 hpi.

Human airway organoids plated in Transwell inserts at ALI for at least 4 weeks were apically inoculated at an MOI of 0.1 and incubated at 37°C and 5% CO. Supernatants were collected at 0, 2, 24, 48, 72, and 96 hpi.

Virus titers were determined by plaque assays as described above.

### Immunoblotting

The absence of accessory proteins in mutants was verified by western blot. Vero E6/TMPRSS2 cells were infected with SARS-CoV-2 deletion mutants at an MOI of 1. At 24 hpi, cells were lysed with 1× LDS Sample Buffer (NuPAGE; Invitrogen). Cell lysates were resolved on a 4%–12% Bis-Tris polyacrylamide gel (NuPAGE, Invitrogen) and transferred to a polyvinylidene difluoride membrane using a Trans-Blot Turbo system (Bio-Rad). Membranes were blocked overnight with 5% milk in TTBS (20 mM Tris-HCl, 150 mM NaCl, 0.1% Tween-20, pH 7.5) at 4°C and hybridized with a primary antibody for 1 hour at room temperature (RT). The primary antibodies used for immunoblotting were mouse monoclonal anti-SARS-CoV-2 N (1:1,000, kindly provided by Ingenasa, Spain), rat anti-ORF6 (1:1,000) (60), sheep anti-ORF7a (1:1,000), sheep anti-ORF7b (1:1,000), sheep anti-ORF8 (1:250) (MRC I PPU, College of Life Sciences, University of Dundee, Scotland), and rabbit anti-β-actin (13E5) (1:2,000; Cell Signaling Technology-4970S). All primary antibodies reacted with horseradish peroxidase (HRP) dye-conjugated secondary antibody and were visualized using the Clarity Western ECL Substrate (Bio-Rad) and the ChemiDoc (Bio-Rad). HRP-conjugated secondary antibodies (1:10,000) were rabbit anti-rat (Sigma-Aldrich-A5795), rabbit anti-sheep IgG (H + L) (Invitrogen-31480), goat anti-mouse IgG (Fc specific) (Sigma-Aldrich-A0168), and goat anti-rabbit (Sigma-Aldrich-A0545).

### *In vivo* infections

Female K18-hACE2 C57BL/6J transgenic mice (strain 2B6.Cg-Tg(K18-ACE2)2Prlmn/J) were purchased from the Jackson Laboratory (Bar Harbor, ME). Mice were housed under pathogen-free conditions and acclimatized to the BSL3 animal facilities in the Animal Health Research Center (CISA/INIA-CSIC) for 7 days prior to infection. Twenty-six-week-old mice were anesthetized with isoflurane and intranasally inoculated with 10^5^ PFU/animal of each recombinant virus diluted in 50 µL of DMEM. Body weight loss and mortality were monitored daily until 10 dpi (*n* = 5 mice per recombinant virus). Mice showing >20% reduction of their initial body weight were euthanized. Twenty-week-old mice were euthanized and necropsied at 1, 2, 3, 4, and 6 dpi (*n* = 4).

For 2D ALI cultures, cells were apically washed three times with AdDF+++ medium prior to infection. Then, cells were apically inoculated at an MOI of 0.1 and incubated for viral adsorption at 37°C. After 2 hours, the inoculum was removed and cells were washed. Then, cells were incubated at 37°C and 5% CO. Samples for RNA extraction was taken at 24 and 72 hpi.

### Competition assay by virus co-infection

Air–liquid airway organoids differentiated in Pneumacult were infected at a 1:1 ratio with SARS-CoV-2-WT and SARS-CoV-2-∆8, at a final MOI of 0.1 in triplicate. Apical washes were performed daily for 4 days and stored at −80°C until further processing. RNA was extracted from the washes and subjected to cDNA synthesis using Superscript IV (Invitrogen). Next, a 1,506- or 1,153-bp region including or not ORF8 was amplified by PCR using Pfu UltraII (Agilent) using the following primers:

Forward: 5′- ACCAAGAGTGTGTTAGAGG-3′; reverse: 5′-GTTCAATCTGTCAAGCAGC-3′.

Conditions for the PCR were as follows: (i) denaturation for 3 minutes at 94°C; (ii) denaturation for 20 seconds at 94°C; (iii) denaturation for 20 seconds at 94°C; (iv) annealing for 20 seconds at 56°C; (v) extension for 90 seconds at 72°C; (vi) steps ii–iv 30 times; and (vii) final extension for 5 minutes at 72°C.

PCR products were purified using PCR purification kit (Qiagen) before Sanger sequencing using the forward primer. Sanger sequences were analyzed using QSVanalyser.

### Processing of mice samples

Lungs and nasal turbinates from euthanized mice were collected to determine viral titers for RNA extraction and histopathological analysis. The left lung lobes were collected for histopathological analysis, while the right lungs were divided into two longitudinal sections (including the three lobes) for viral titration and RNA analysis, respectively. Samples were homogenized in 1 mL of phosphate-buffered saline (PBS) (Sigma-Aldrich) with 100 IU/mL penicillin (Sigma-Aldrich), 0.1 mg/mL streptomycin (Sigma-Aldrich), 50 µg/mL gentamicin (Sigma-Aldrich), and 0.5 µg/mL amphotericin B (Sigma-Aldrich) using a gentleMACS dissociator (Miltenyi Biotec, USA) for lung tissues, or the BeadBug 6 microtube homogenizer (Sigma-Aldrich) and metal beads lysing matrix S tubes (MP biomedicals, USA) for nasal turbinates. RNA from homogenized mouse lungs was purified using the RNeasy kit (Qiagen, Germany) following the manufacturer’s specifications.

### Histopathological analysis

Left lung lobes from mice were fixed in 10% zinc formalin (Sigma-Aldrich) for 48 hours. After the fixation period, samples were processed and embedded in paraffin blocks that were then sectioned at 4-µm thickness on a microtome, mounted onto glass slides, and stained with hematoxylin and eosin (H&E). Lung sections were evaluated using an Olympus BX43 microscope. To assess the character and severity of histopathological lesions, lung inflammation scoring parameters based on the previous reports on SARS-CoV-2 infection in mouse models were used (36). The histopathological parameters evaluated were hemorrhages, edema, septal thickening, alveolar damage, hyaline membranes, and inflammatory cell infiltration in alveoli. The histopathological parameters were graded following a semi-quantitative scoring system as follows: (0) no lesion; (1) minimal lesion; (2) mild lesion; (3) moderate lesion; and (4) severe lesion. The cumulative scores of histopathological lesions provided the total score per animal. In each experimental group, the individual scores were used to calculate the group average.

### Immunohistochemistry

The detection of macrophages was assessed in paraffin sections. Antigen retrieval was done by microwaving sections in citrate buffer (10 mM, pH 6.0, Sigma-Aldrich) for 5 minutes at 100°C. After endogenous peroxidase blocking with 3% H_2_O_2_ for 10 minutes, slices were blocked again with 10% Normal Goat Serum for 1 hour at RT. Primary antibodies [1:100, Rabbit-anti-F4/80 (no. D2S9R) XP, Cell Signaling Technology or Normal Rabbit IgG Control (AB-105-C; R&D systems)] were incubated in incubation buffer (0.1% BSA in PBS) for 1 hour at RT. Then, sections were incubated with a secondary antibody conjugated with HRP (1:100, Goat-anti-rabbit IgG-HRP, P0448; DAKO) for 1 hour at RT. Finally, HRP activity was revealed by incubating the slides for 20 minutes at RT in a substrate containing 1.5 mL of 3-amino-9-ethyl-carbazole (AEC; Sigma-Aldrich) dissolved in *N*, *N*-dimethylformamide (DMF; Sigma-Aldrich) (17 mg/mL) and 30 µL of H_2_O_2_ 30% in 60 mL of NaAc buffer, pH 5.0, followed by washing step in PBS. The use of AEC results in a bright red precipitate.

Staining on two sections of each tissue was performed. The evaluation was performed by light microscopy by two independent observers blinded to the fixation procedure. The evaluation included the macrophage count in lesions, including three mice per conditions.

### Monocyte isolation and maturation to monocyte-derived macrophages (MDMs)

Heparinized blood was obtained from healthy volunteers after informed consent. Monocytes were isolated from peripheral blood mononuclear cells (PBMCs) using monocyte-positive selection by monoclonal CD14 antibody (mouse IgG2a isotype)–conjugated microbeads (Miltenyi Biotec, Germany) according to the manufacturer’s protocol. Monocytes were cultured in flasks in complete maturation media (RPMI-1640 with 10% fetal calf serum [FCS], 100U/100 µg/mL penicillin/streptomycin [Thermo Fisher Scientific], 50 ng/mL granulocyte–macrophage colony‐stimulating factor [Peprotech]) for 7–10 days for MDM differentiation. Media were changed every 3 days.

### Macrophage incubation with conditioned media from infected organoids

Conditioned media from organoid-derived human airway cells either mock-infected or infected with viruses at an MOI 0.1 were collected at 24 and 72 hpi from the bottom part of the insert. After confirming that the infectious virus was not detected in the conditioned media, MDMs were incubated with 100 µL of conditioned media from each condition for 24 hours. Images were taken with a Zeiss Primovert microscope using Zen software (Zeiss) before and after MDM incubation. The percentage of activated macrophages after incubation with the supernatant from infected cells was quantified with the ImageJ v2.1.0 software, using three independent images per condition.

### RNA analysis by RT-qPCR

Total cDNA was synthesized using 100 ng of purified RNA as template, the High-Capacity cDNA Reverse Transcription kit (Applied Biosystems, USA), and random hexamers. Two microliters of cDNA were analyzed by real-time qPCR for quantification of genomic viral RNA and host innate immune response gene expression. qPCRBIO Probe Mix No-Rox MasterMix (PCR Biosystems, United Kingdom) and validated Taqman assays (Thermo Fisher Scientific, USA) specific for IFN-β (Mm00439552_s1), IFN-λ3 (Mm00663660_g1), ISG15 (Mm01705338_s1), MX1 (Mm00487796_m1), IFIT1 (Mm07295796_m1), OAS1 (Mm00836412_m1), XAF1 (Mm01245815_m1), TNF-α (Mm00443258_m1), CXCL10 (Mm00445235-m1), IL-6 (Mm00446190_m1), CCL2 (Mm00441242_m1), CCL7 (Mm00443113_m1), and CXCL11 (Mm00444662_m1) were used for animal samples. On the other hand, IFN-β (Hs01077958_s1), IFN-λ (Hs00820125_g1), ISG15 (Hs01921425_s1), IFIT1 (Hs03027069_s1), OAS1 (Hs00973635_m1), TNF-α (Hs00174128_m1), CXCL10 (Hs00171042_m1), IL-6 (Hs00174131_m1), CXCL11 (Hs00171138_m1), and CCL7 (Hs00171147_m1) for organoid samples. A custom Taqman assay previously described was used to quantify SARS-CoV-2 genomic RNA (61). The levels of rRNA 18S (Mm03928990_g1), β-actin (Hs02742610_g1), and SARS-CoV-2 genomic RNA were used as an internal control for normalization. QuantStudio 5 real-time PCR system (Applied Biosystems) and QuantStudio software (Version 1.5.2) were used to acquire and analyze data, respectively. Relative quantifications were performed using the 2^-∆∆CT^ method (62).

### Statistical analysis

Student’s *t*-test was used to analyze differences in mean values between groups. All results were expressed as means ± SEM. *P* values < 0.05 (*), <0.01 (**), <0.001 (***), and <0.0001 (****) were considered statistically significant.

Survival analysis was interpreted by log-rank (Mantel–Cox) test. Survival of all groups were compared with the survival of mock-infected group. Significant values were indicated as asterisks (*P < 0.05; **P < 0.01). Statistical analysis was performed by GraphPad Prism, version 9.
